# Supplementary material for: Explaining the emergence of complex networks through log-normal fitness in a Euclidean node similarity space
Source: Sci Rep. 2021 Jan 21;11:1976. doi: 10.1038/s41598-021-81547-3 (PMC7820353; doi:10.1038/s41598-021-81547-3)
Supplement: Supplementary file 1 — Supplementary Information. [file 41598_2021_81547_MOESM1_ESM.pdf]

Explaining the emergence of complex networks through log-normal fitness in a Euclidean node similarity space

Keith M. Smith

Supplementary material

This supplementary material contains supporting evidence, figures and tables for the article “Explaining the emergence of complex networks through log-normal fitness in a Euclidean node similarity space”. The contents are as follows:

- Section I describes additional model experiments carried out to understand what to expect from the topology of our model and modelling algorithm
- Section II provides supporting results from the modelling of real-world network topologies in the main article
- Section III provides details regarding the optimisation of surface inversion factor using skewness of the inverted link weight distribution
- Section IV provides details of the complementary surface inversion analysis of an fMRI weighted network
- Section V provides the tables of nearest neighbours discovered in the global city network experiment

Section I: Model experiments

This section details exploratory analysis of surface-depth models. In part A we look at topological properties of the networks using common network metrics. Particularly, we are interested in how the model behaves by increasing the number of dimensions of the depth factor and whether the topological properties exhibited match general expectations of the topology of real-world networks. In part B, we go on to analyse the typical degree distributions of the model at different densities. We are particularly interested in how the model behaves with varying the shape parameter, σ, of the surface factor. Using statistical tests, we check to what extent the degree distributions of the model resemble power-laws and log-normal distributions at different densities. Part C describes a validation of the algorithm for modelling real-world networks using the surface-depth model by subjecting the algorithm to surface-depth models themselves and comparing the true parameters with those estimated by the algorithm.

A. Topology of surface-depth models

Here, we explore the topology of surface-depth models using common network metrics. We generated realisations of $G_{s-d}(q, 0.1)$, with $q = 1,2,3,\dots,100$, for networks of size 256 and binarised these networks at 10% density. Here, binarisation refers to the process of selecting the edges with the largest T% weights as existent edges in the network and the rest as non-existent. We did the same analysis for random geometric graphs with q dimensions. For each q, 50 realisations of surface-depth models and random geometric graphs were generated and their topologies characterised by C, E, V, Q and r (see Methods & Materials of the main article for details of metrics). Figure A shows the mean results over these realisations. By adding the log-normal surface factor to random geometric graphs, we see that the global clustering and degree variance are increased. C doubles in size while degree variance shows an even greater appreciation with the addition of the surface factor. These two characteristics jointly help to describe the creation of a rich-club which is determined by the nodes with the largest values of the surface factor distribution. The expected value $d_{ij}(s_i + s_j)$ is large when s_i and s_j are both large. Generalising this, the k largest nodes will have the largest $k(k-1)$ values coming from the surface factor $(s_i + s_j)$.

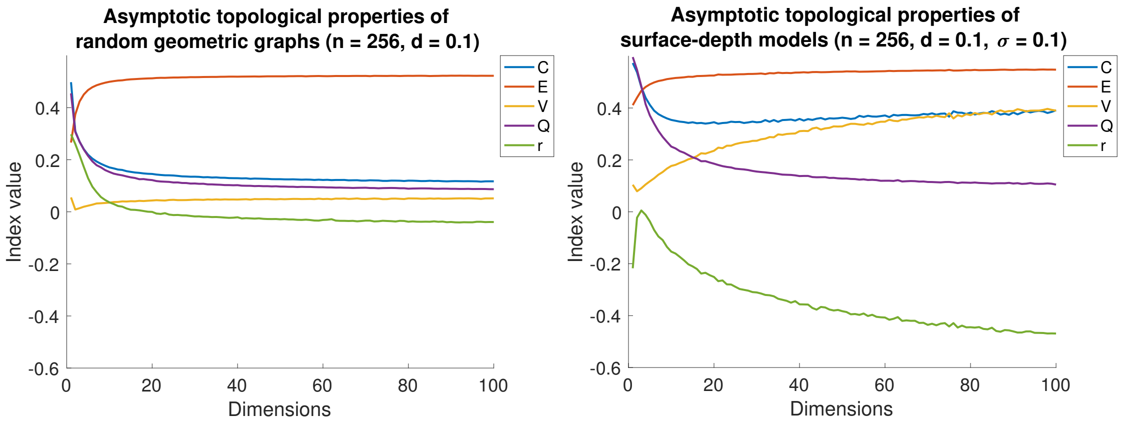

Figure A. Comparison of network index values (see legend) of random geometric graphs and surface-depth models over varying dimension. Topologies appear to be asymptotic with respect to dimension. Abbreviations: n- network size, d- network density, σ- surface factor parameter, C- clustering coefficient, E- global efficiency, V- normalized degree variance, Q- modularity, r- assortativity.

On the other hand r is pushed down, going from just below zero to a large negative value closer to -0.4, for large q. This appears very symmetric with the behaviour of V and is explained by the fact that, alongside high degree nodes prominently connecting to other high degree nodes, the large number of small degree nodes are also more likely to connect to high degree nodes. Since there are generally far larger numbers of small degree nodes, node-node degree correlations would, on average, be negative. Interestingly, E and Q maintain similar values after applying the surface factor, although Q is increased for small values of q. This tells us, in this particular experiment, that the network efficiency and tendency to form communities is similar between random geometric graphs and surface-depth models. These are all positive signs for our theory. Non-bipartite complex networks are generally found to have large clustering [1], high degree heterogeneity [2] and disassortativity [3].

B. Degree distributions of surface-depth models

Next, we demonstrate that surface-depth models exhibit power-law distributions at sparse densities and log-normal distributions in larger densities. Denoting $P(k)$ as the fraction of nodes of degree k, power-laws were fitted by doing a linear regression using least squares on $ln(P(k))$ against $ln(k)$. The best-fit γ was then recovered by $exp(1/b_1)$ where b_1 is the coefficient of the first power of $ln(k)$ in the linear regression. The regression was done on the largest range of $P(k)$ found without any 0s (as $ln(0)$ is undefined). Once the line-of-best-fit was found, these were compared against the actual log-log-distribution using Kolmogorov-Smirnov (KS) two sample tests. These same tests were used in [4]. Notably, Voitalov, et al. [5] criticized KS tests for being parametric and for relying on *p*-values. However, two-sample KS tests are not parametric and are very useful for comparing the shapes of two distributions. Moreover, if the power-law is anywhere near a good fit, it should definitely have a non-significant *p*-value.

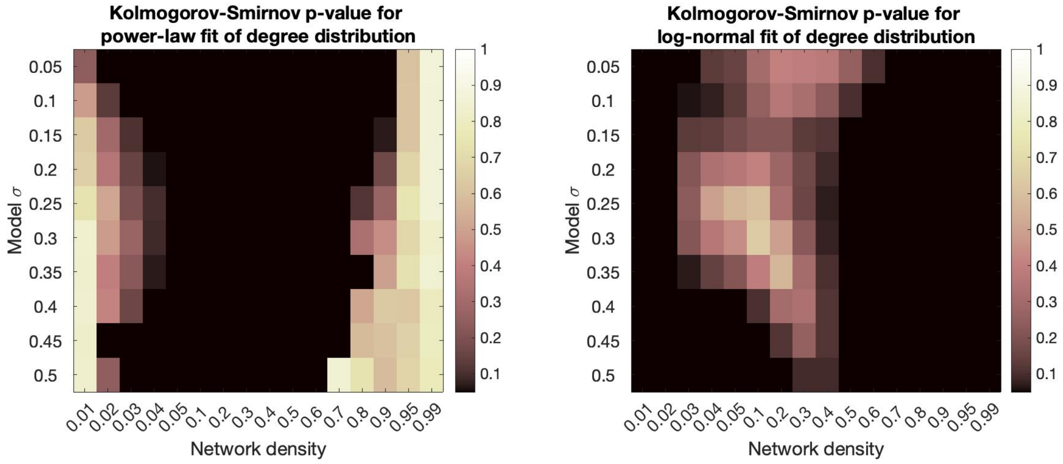

Figure B. Left shows goodness-of-fit of power-laws to the degree distributions of surface-depth models (n=1000, q=4). Right shows goodness-of-fit of log-normal distributions to the degree distributions of surface-depth models. In both, network density is plotted against models with different surface factor shape parameter, σ. Blacked out elements of the matrices indicate that the null hypothesis— that the two distributions are the same— was rejected at the $\alpha = 0.05$ level. Brighter elements indicate more confidence in the similarity of the distributions. Results are the median over 100 iterations.

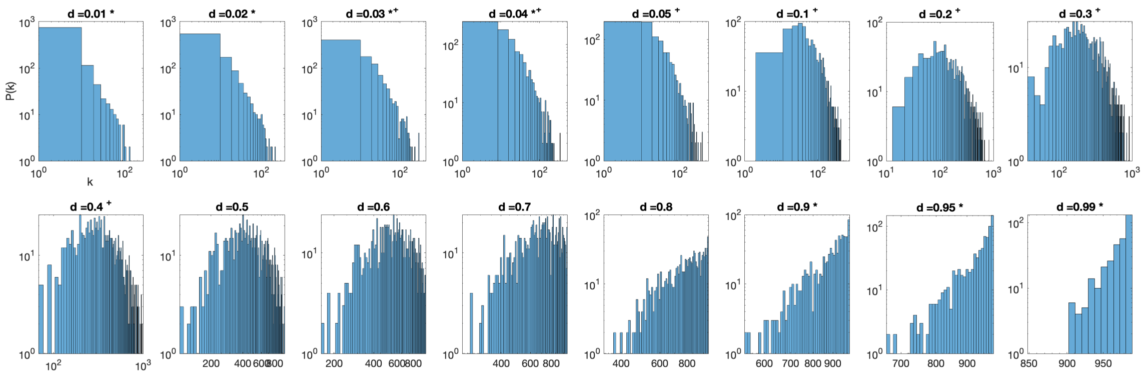

*Figure C. Example of degree distributions obtained from surface-depth models (n = 1000, σ = 0.2, q = 4) binarised at different densities, d. A * in the title indicates that the null hypothesis of the KS test for power-law fit was not rejected (i.e. the distribution cannot be said to be different from a power-law), while + indicates that the null hypothesis of the KS test for log-normal fit was not rejected, as per Figure B. All axes are as described in the bottom left plot—degree, k, against number of nodes with that degree.*

Log-normality was tested by assessing whether the distribution of the log of degrees, $ln(k)$, could be distinguished from a normal distribution. Note, estimates of the μ and σ parameters of a best-fit log-normal distribution can be taken as the mean and standard deviation of $ln(k)$, since this is precisely how μ and σ of the log-normal distribution are defined (i.e. as the mean and standard deviation of a normal distribution). Testing the log-normality of a distribution is equivalent to testing normality of the log of the distribution, so one could use a test for normality such as the powerful Shapiro-Wilk's test. However, the degree distribution is discrete, taking integer values, so comparing against a similarly discretized log-normal distribution is more meaningful. Therefore, 1000 random samples of the log-normal distribution with $\mu = < ln(\boldsymbol{k}) >$ and $\sigma = \sqrt{var(ln(\boldsymbol{k}))}$ were generated. To discretise this in line with degree distributions, these were then rounded to the nearest integer and a two sample KS test was conducted between the degree distribution of the model and the randomly sampled and discretized log-normal data.

Both sets of tests were done on surface-depth models with 1000 nodes and $q = 4$ depth factor dimensions. The surface factor parameter σ varied from 0.05 up to 0.5 in steps of 0.05 to assess the effect of the right-skew of the surface factor on the degree distribution. Network densities were selected at 0.01, 0.02, 0.03, 0.04, 0.05, 0.1, 0.2, 0.3, 0.04, 0.5, 0.6, 0.7, 0.8, 0.9, 0.95 and 0.99, allowing more analysis at more sparse densities to better cover sparse network power-laws. For each model and each density, one hundred iterations were run and median *p*-values reported in Figure B, with examples of the degree distribution at each density for a model with $\sigma = 0.2$ in Figure C. The null hypothesis, that the two observed samples come from the same underlying distribution, was not rejected if the *p*-value was greater than 0.05. From this, we can see that degree distributions could not be distinguished from power-laws at densities of up to 0.04. Beyond this, distributions appeared log-normal from around 0.04 up to 0.4. Degree distributions for densities larger than 0.9 again tended to be indistinguishable from power-laws, but with positive exponents.

C. Validation of Algorithm 1

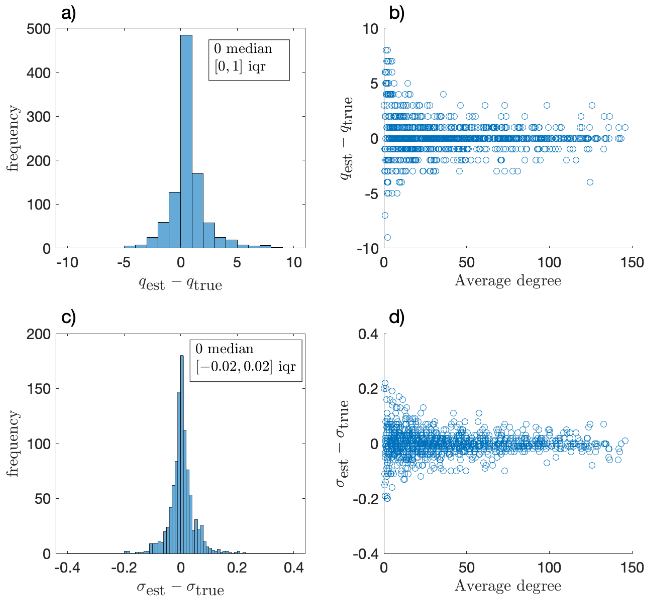

Figure D. Statistics of algorithm validity: a) The distribution of errors of the estimated depth factor, q_{est}, from the true depth factor, q_{true}. b) The error in q_{est} improves in models with greater average degree. c) The distribution of errors of the estimated surface factor, σ_{est}, from the true surface factor, σ_{true}. d) The error in σ_{est} improves in models with greater average degree.

Here, we describe an experiment run to assess the accuracy of algorithm 1 which models networks with surface-depth models. This was done by running algorithm 1 against randomly generated surface-depth models themselves. In this way, the accuracy of parameters of the surface depth model returned by the algorithm could be determined by the error from the true parameters of the randomly generated model. To efficiently incorporate a wide range of network sizes and densities and parameters of the model, 1000 randomly parameterised G_{s-d} models were generated with size $n \sim U(\llbracket 25{,}500 \rrbracket)$ (i.e. uniformly among the integers from 25 to 500), density $P \sim U([0.001{,}0.3])$, depth factor $q \sim U(\llbracket 1{,}10 \rrbracket)$ and surface factor $\sigma \sim U(\{0.02{,}0.04, \ldots, 0.4\})$ (i.e. uniformly on the among the set of numbers $\{0.02{,}0.04, \ldots, 0.4\}$). The algorithm is the same as algorithm 1 except that the averaging over 20 iterations was removed so that a large number of different models could be analysed at speed. The results showed that the interquartile range (i.e. 50%) of the distribution of the error of the algorithm was within 1 dimension of the true dimension for the depth factor and within 0.02 of σ of the surface factor, as shown in the text inset in Figure D (a) and (c). Furthermore, the errors for both factors were seen to improve with increasing average degree of the networks. This is unsurprising since large average degree implies larger number of nodes, larger number of links or both, hence indicating more information.

Spearman correlations between the true parameters and the errors of the algorithm show notable positive correlations. The correlation for q was 0.3085 with a p-value of 1.73×10^{-23}. This means that if the model estimates a large number of dimensions in the depth factor it is more likely to have greater error. The same tendency was true with σ. The Spearman correlation between the estimated σ and the error from the true value is 0.4004 with a p-value of 8.54×10^{-40}. This implies that a larger σ from the algorithm is more likely to have a greater error from the true value. This is likely because the tail of the log-normal distribution plays a significant role in determining the variance of the distribution, while being less likely to be accurately represented in a finite random sample.

Section II: Approximating real-world networks

The comparisons of indices of 10 of the networks from the network repository are shown in Figure E. It is clear that the index values attained vary quite widely across real world networks, yet the model clearly adapts appropriately to each topology. Further, these are compared with topologies of the geometric random graph with the same number of dimensions as the best-fit surface-depth model (yellow markers) and networks made up solely from the surface factor of the best-fit model applied to Erdös-Rényi random graphs (green markers). Therefore, it is clear that the adaptation of the model to each topology cannot be explained by bias in network indices to number of nodes and edges.

Complementing Fig 3 in the main article, Fig F here shows the degree distributions of all ICON networks and their best-fit models. Most degree distributions are well approximated by the model. Particularly badly fitting model degree distributions are seen in the genetic and digital circuit networks as well as a minority of protein interaction networks. We conjecture that better modelling of the depth factor in these networks, with detailed considerations of the nature of latent variables, would provide better fits for these networks. For example, we may suppose that some of the variables are categorical, rather than continuous. Some variables are also more likely to take on other distributions than uniform, such as normal or, indeed, log-normal. Furthermore, certain factors are likely to be more important than others, which could be accounted for by providing weight coefficients in the distance function.

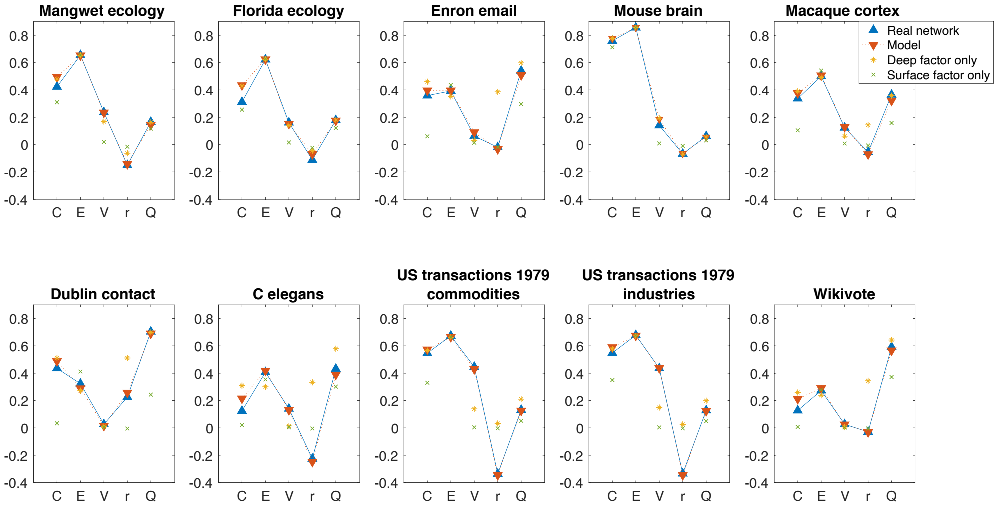

Figure E. Network index values of real-world networks and surface-depth models. Also plotted are the networks stemming from depth and surface factors separately.

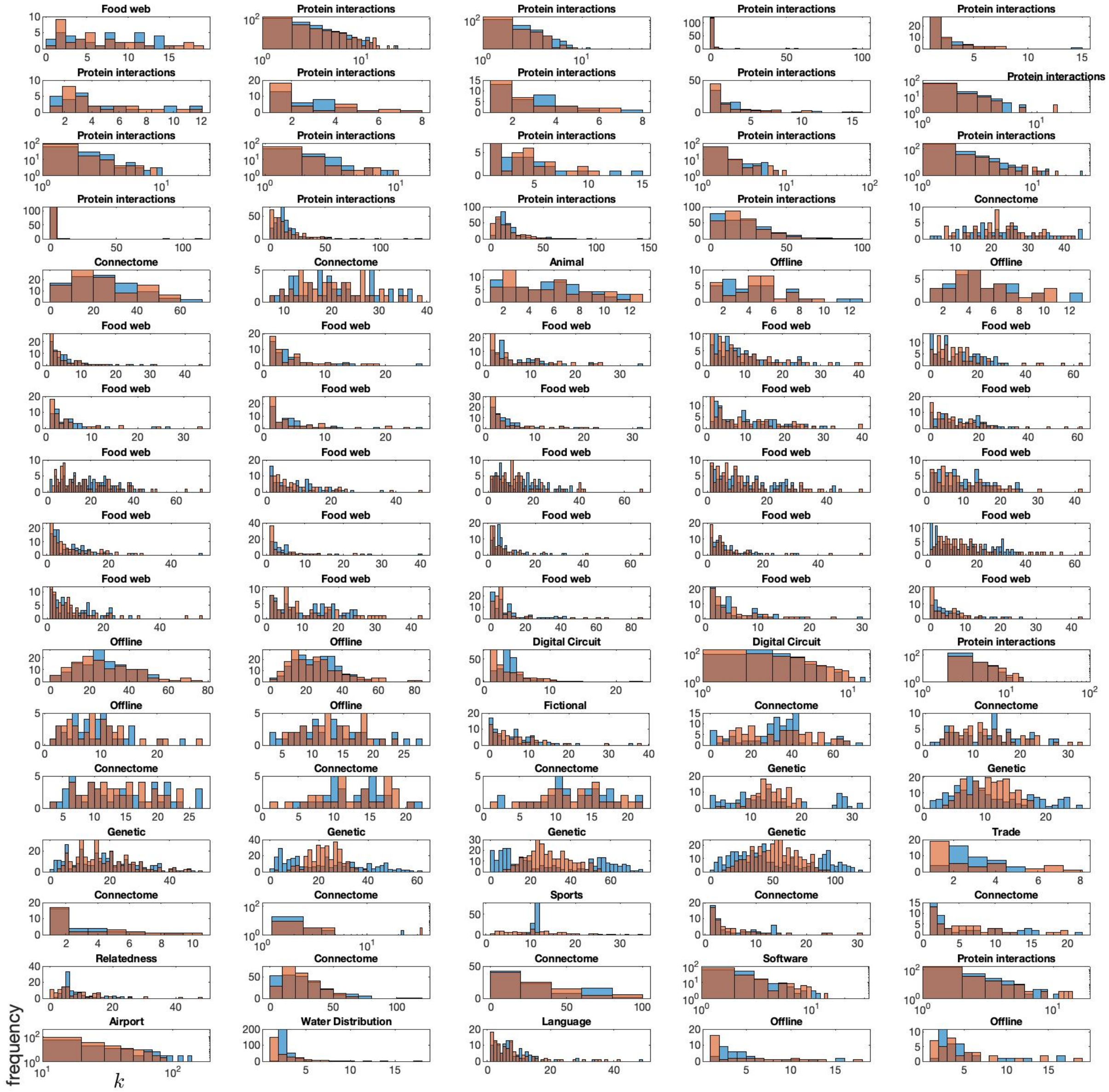

Figure F. Comparison of degree distributions of real-world networks (blue) and single realisations of their best-fit surface-depth models (red), with titles indicating network class. Axes are as in bottom left panel (x-axis is degree, k; y-axis is frequency of those degrees in the network). The general shapes of distributions are well approximated in most instances. Only some large disparities are visible, particularly in the genetic networks and the sports network.

Section III: Skewness of distances in q-dimensional Euclidean space

Here we demonstrate the distributions (with skewness) of randomly selected distances in Euclidean space using a large number of simulated observations. Five hundred thousand Euclidean distances between pairs of randomly selected samples of 2D, 3D, 5D and 20D Euclidean space were computed and the skewness of the distributions, γ_1, were analysed, Fig G. The skewness of these distributions is negligible for $q > 2$, while the value for $q = 2$ is still fairly small. Analytically, the limit of these distributions as $q \to \infty$ is the normal distribution, as discussed in the main article.

We analysed the validity of this approach by studying the correlations of weights between true and estimated depth factors from our model. This was done using a surface-depth model with n = 256, a 4D Euclidean depth factor (i.e. q = 4) and log-normal surface parameter σ varying from 0.01 up to 0.25 in steps of 0.01. 100 iterations of the model for each surface parameter value were generated. Surface inversion attempts were then implemented on these generated models using all possible surface parameter values again from 0.01 up to 0.25 in steps of 0.01. The weights of the estimated depth factors were then analysed to see if smaller values of skewness matched up with higher correlations between estimated and true depth factors, taking averages over each set of 100 iterations. The results are shown in Fig H, showing a clear inverse relationship between correlation of true and estimated depth factors and skewness of estimated depth factor. All in all, this substantiates the minimisation of skewness of the estimated depth factor as a meaningful optimisation argument for surface factor estimation.

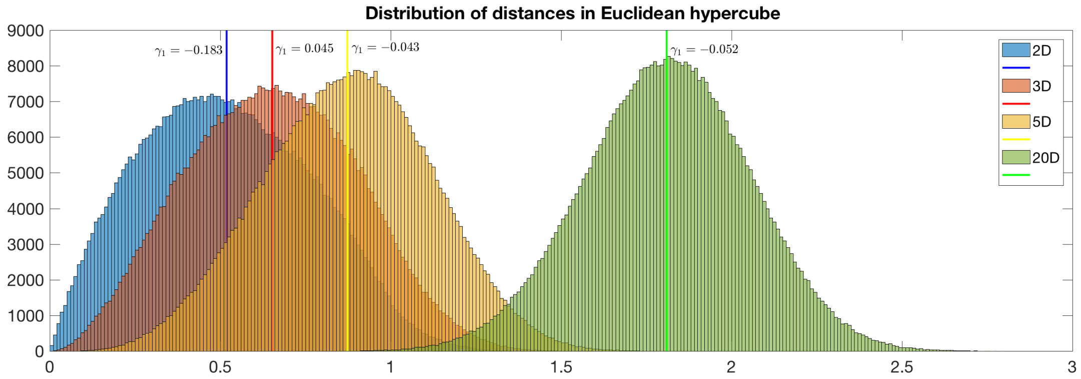

Figure G. Distributions of distances in Euclidean spaces. Mean values are shown by the bold coloured lines, while the skewness is written above each distribution

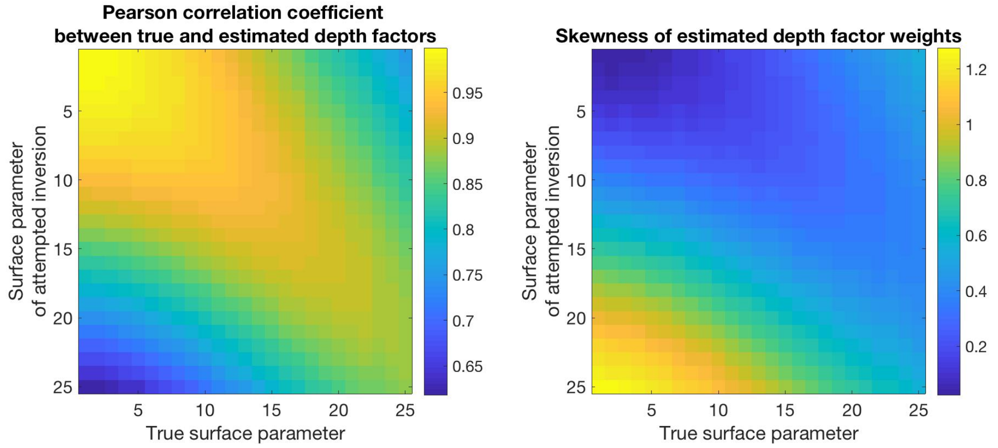

Figure H. The higher the correlation between true and estimated depth factor weights (left), the smaller the skewness of those estimated depth factor weights tends to be

Section IV: Weighted fMRI network analyses

For the surface inversion efforts on the fMRI network, first, 1000 iterations of the optimal log-normal surface factor (following algorithm 2 in the main article) were computed for the fMRI network. Then, 5NN binary networks were constructed for both the original network and the 1000 estimated depth factors. The 5NN binary network contains unweighted links between each node and their five nearest neighbours. Alongside this, the 5NN brain geometry graph was constructed using the Euclidean distances between brain region co-ordinates. The geometric overlap, GO, was then computed for the original network and the model realisations as the proportion of links in the 5NN networks which also existed in the 5NN brain geometry graph. That is, for two network adjacency matrices **A** and **B** with entries A_{ij} and B_{ij}, respectively, and m links:

$$GO(\boldsymbol{A},\boldsymbol{B}) = \frac{1}{2m}\sum_{i,j=1}^{n} match(A_{ij} + B_{ij})$$

Where

$$match(x) = \begin{cases} 1 \ if \ x = 2 \\ 0 \ otherwise \end{cases}$$

Louvain's modularity algorithm was then used to determine network modules. Since this does not necessarily result in the same modules, this was done 1000 times over the real network. Examples of the community detection on brain regions, as per their locations on the brain surface, are shown in Figure I. For the modules, three metrics were devised for assessing their geometric qualities. Firstly, we used the Normalised Mutual Information (NMI) [6]

$$NMI = \frac{I(X;Y)}{\sqrt{H(X)H(Y)}}$$

For discrete random variables X and Y, where

$$I(X;Y) = H(X) - H(X|Y)$$

is the mutual information between X and Y are random discrete variables, $H(X)$ is the entropy of X and $H(X|Y)$ is the conditional entropy of X given Y. NMI was computed between the modules detected in the estimated depth-factor and the geometric graph as well as between the modules detected in the fMRI network and the modules from the geometric graph for comparison. Then, we computed the largest distance between any two nodes in the same module and same hemisphere and averaged this over all modules and both hemispheres, i.e. for weighted adjacency matrix **W** with *N* modules $c_1, c_2, \dots, c_N$, where $c_1^L, c_2^L, \dots, c_N^L$ are the corresponding members of those modules in the left hemisphere and $c_1^R, c_2^R, \dots, c_N^R$ in the right hemisphere, we obtain the metric

$$LD = \frac{1}{2}\left(\frac{1}{N}\sum_{k=1}^{N} \max_{\mathrm{i,j \in c_k^L}} w_{ij} + \frac{1}{N}\sum_{k=1}^{N} \max_{\mathrm{i,j \in c_k^R}} w_{ij}\right)$$

Lastly, module symmetry was computed as the fraction of regions for which their hemispherically symmetric counterpart was in the same module. The results are plotted in Fig J. The estimated depth factor showed a very strong and clear increase in geometric overlap from the original network where every single estimation showed greater overlap than the original. NMI and module symmetry were both significantly greater in the depth factor (Wilcoxon rank sum tests $p = 8.01 \times 10^{-23}$, $p = 9.83 \times 10^{-26}$, respectively, with Cohen's d = 0.4485, d = 0.4304, respectively) while average largest distance within modules was significantly less (Wilcoxon rank sum test $\mathrm{p} = 1.89 \times 10^{-14}$, Cohen's d = -0.3162). All of these confirm our expectation that the depth factor consists of similarities between nodes once the node fitness tendencies of the surface factor are removed/dampened.

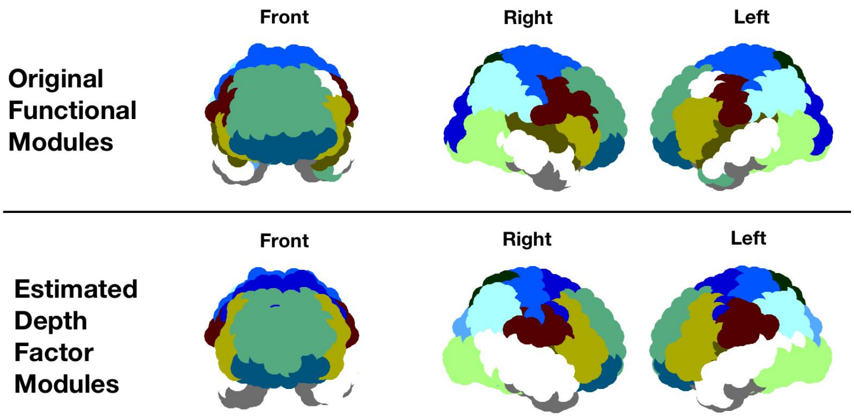

Figure I. Examples of modules obtained from the original fMRI network and the estimated depth factor. Each circle is a node and the circles overlap to aid in illustrating where these regions are located on the surface of the brain. The different colours are arbitrary and relate to the different communities detected using the community detection algorithm. The depth factor shows significantly greater symmetry between hemispheres and a greater overlap with the geometric graph based on the coordinates of brain regions.

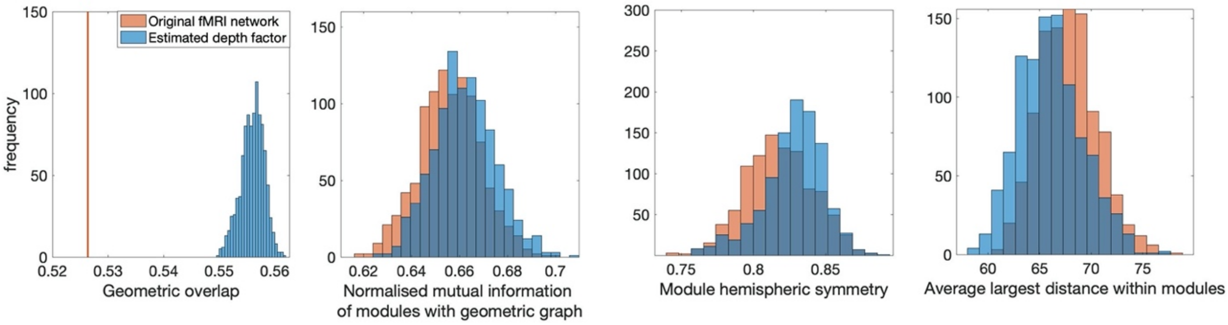

Figure J. Metrics of geometric consistency of the fMRI network and its estimated depth factor over 1000 iterations of depth factor realisation and module partitioning

Section V: Tables of nearest neighbours in the World City Network

Here presented in Tables A, B and C are the tables of the five nearest neighbours for each city in the world city network (described in the main document in the Real-world Network Data subsection of the Methods and Materials section) and it's estimated depth factors using both a tuned log-normal distribution and the weighted degree distribution. Each table is followed by some summary statistics relating to the appearance of the top five cities with largest weighted degree and the number of proximal relations found between each city and these top five largest degree cities. The following categories describe the groups of proximal cities:

North America: Atlanta, Boston, Chicago, Dallas, Houston, Los Angeles, Mexico City, Miami, Minneapolis, Montreal, New York, San Francisco, Toronto, Washington D.C.

South America: Buenos Aires, Caracas, Santiago, Sao Paulo

Europe: Amsterdam, Barcelona, Berlin, Brussels, Budapest, Copenhagen, Dusseldorf, Frankfurt, Geneva, Hamburg, Istanbul, London, Madrid, Milan, Moscow, Munich, Paris, Prague, Rome, Stockholm, Warsaw, Zurich

East Asia and Oceania: Tokyo, Hong Kong, Sydney, Singapore, Taipei, Melbourne, Jakarta, Seoul, Bangkok, Manila, Kuala Lumpur, Beijing, Shanghai, Osaka

Mexico City and Miami were also considered proximal to South America

Table A. Five Nearest Neighbours (NN) for each node from the reconstructed depth factor after surface inversion using a tuned log-normal distribution (Green indicates globally proximal neighbour)

City	NN1	NN2	NN3	NN4	NN5
**London**	New York	Paris	Hong Kong	Los Angeles	Tokyo
**New York**	Los Angeles	Hong Kong	Washington	Tokyo	Paris
**Paris**	Brussels	Frankfurt	Hong Kong	Los Angeles	Madrid
**Tokyo**	Hong Kong	Frankfurt	Singapore	New York	Zurich
**Hong Kong**	Singapore	Los Angeles	Taipei	Frankfurt	New York
**Sydney**	Hong Kong	Taipei	Melbourne	Toronto	Seoul
**Singapore**	Hong Kong	Taipei	Zurich	Bangkok	Frankfurt
**Milan**	Madrid	Frankfurt	Zurich	Sao Paulo	Toronto
**Frankfurt**	Zurich	Milan	Hong Kong	Madrid	Paris
**Toronto**	Amsterdam	Melbourne	Madrid	Dusseldorf	Zurich
**Madrid**	Milan	Barcelona	Santiago	Sao Paulo	Frankfurt
**Brussels**	Washington	Madrid	Amsterdam	Paris	Milan
**San Francisco**	Washington	Los Angeles	Chicago	Boston	Hong Kong
**Los Angeles**	Washington	San Francisco	New York	Hong Kong	Chicago
**Zurich**	Frankfurt	Santiago	Milan	Madrid	Mexico City
**Mexico City**	Zurich	Sao Paulo	Frankfurt	Buenos Aires	Santiago
**Chicago**	Washington	Dallas	Los Angeles	San Francisco	Frankfurt
**Sao Paulo**	Santiago	Madrid	Zurich	Milan	Caracas
**Amsterdam**	Barcelona	Melbourne	Dusseldorf	Stockholm	Toronto
**Taipei**	Bangkok	Seoul	Shanghai	Jakarta	Kuala Lumpur
**Melbourne**	Amsterdam	Dusseldorf	Stockholm	Bangkok	Toronto
**Jakarta**	Taipei	Bangkok	Seoul	Manila	Miami
**Seoul**	Shanghai	Taipei	Kuala Lumpur	Jakarta	Bangkok
**Washington**	Los Angeles	San Francisco	Dallas	Chicago	Boston
**Dusseldorf**	Hamburg	Copenhagen	Boston	Amsterdam	Melbourne
**Moscow**	Warsaw	Washington	Prague	Miami	Buenos Aires
**Stockholm**	Copenhagen	Amsterdam	Warsaw	Hamburg	Dusseldorf
**Bangkok**	Shanghai	Beijing	Kuala Lumpur	Taipei	Manila
**Santiago**	Caracas	Buenos Aires	Barcelona	Sao Paulo	Zurich
**Buenos Aires**	Santiago	Caracas	Zurich	Miami	Manila
**Johannesburg**	Copenhagen	Seoul	Shanghai	Montreal	Stockholm
**Barcelona**	Amsterdam	Santiago	Madrid	Rome	Bangkok
**Dallas**	Washington	Atlanta	Chicago	Montreal	Boston
**Montreal**	Boston	Copenhagen	Dusseldorf	Hamburg	Dallas
**Manila**	Bangkok	Taipei	Jakarta	Shanghai	Beijing
**Warsaw**	Prague	Moscow	Budapest	Stockholm	Istanbul
**Kuala Lumpur**	Shanghai	Bangkok	Seoul	Taipei	Manila
**Prague**	Warsaw	Istanbul	Budapest	Stockholm	Moscow
**Caracas**	Santiago	Miami	Buenos Aires	Sao Paulo	Zurich
**Budapest**	Prague	Warsaw	Istanbul	Miami	Stockholm
**Beijing**	Bangkok	Shanghai	Taipei	Seoul	Manila
**Geneva**	Taipei	Santiago	Seoul	Manila	Zurich
**Miami**	Washington	Caracas	Jakarta	Santiago	Buenos Aires
**Boston**	Dusseldorf	Houston	Washington	Montreal	Istanbul
**Istanbul**	Prague	Copenhagen	Boston	Budapest	Stockholm
**Copenhagen**	Hamburg	Stockholm	Dusseldorf	Istanbul	Johannesburg
**Hamburg**	Copenhagen	Munich	Dusseldorf	Berlin	Atlanta
**Rome**	Munich	Barcelona	Osaka	Dallas	Berlin
**Munich**	Hamburg	Berlin	Copenhagen	Rome	Dusseldorf
**Houston**	Boston	Washington	Dallas	Johannesburg	Geneva
**Shanghai**	Kuala Lumpur	Bangkok	Seoul	Taipei	Beijing
**Berlin**	Munich	Hamburg	Atlanta	Rome	Washington
**Atlanta**	Dallas	Hamburg	Washington	Osaka	Copenhagen
**Osaka**	Atlanta	Hamburg	Munich	Rome	Berlin
**Minneapolis**	Atlanta	Hamburg	Copenhagen	Washington	Berlin

Appearances of top 5 weighted nodes as five nearest neighbours: London- 0 (0); New York- 4 (1); Paris- 4 (3); Tokyo- 2 (0); Hong Kong- 9 (3). In total, this makes 10.56% of the entire closest five relations (expected: 9.27%).

Proximal relations: 180— 3.89% of which are due to the top 5.

All cities except London, Sydney and Minneapolis appear as nearest-neighbours.

Table B. Five Nearest Neighbours (NN) for each node from the reconstructed depth factor after surface inversion using weighted degree distribution (Green indicates globally proximal neighbour)

City	NN1	NN2	NN3	NN4	NN5
**London**	New York	Paris	Los Angeles	Hong Kong	Tokyo
**New York**	London	Los Angeles	Hong Kong	Tokyo	Paris
**Paris**	London	New York	Hong Kong	Tokyo	Brussels
**Tokyo**	New York	London	Hong Kong	Paris	Singapore
**Hong Kong**	New York	London	Singapore	Tokyo	Paris
**Sydney**	London	New York	Hong Kong	Paris	Singapore
**Singapore**	London	New York	Hong Kong	Tokyo	Paris
**Milan**	New York	London	Madrid	Tokyo	Paris
**Frankfurt**	New York	London	Paris	Tokyo	Hong Kong
**Toronto**	London	Sydney	New York	Paris	Madrid
**Madrid**	London	Milan	Paris	New York	Tokyo
**Brussels**	Paris	London	New York	Madrid	Milan
**San Francisco**	New York	London	Washington	Los Angeles	Hong Kong
**Los Angeles**	New York	London	Washington	San Francisco	Hong Kong
**Zurich**	London	New York	Frankfurt	Tokyo	Singapore
**Mexico City**	Tokyo	New York	London	Hong Kong	Zurich
**Chicago**	New York	Washington	London	Los Angeles	San Francisco
**Sao Paulo**	Madrid	Tokyo	London	Milan	New York
**Amsterdam**	Toronto	Sydney	Paris	Madrid	Brussels
**Taipei**	Hong Kong	Singapore	New York	London	Sydney
**Melbourne**	Sydney	London	Toronto	Amsterdam	New York
**Jakarta**	Paris	London	Sydney	New York	Taipei
**Seoul**	Sydney	London	Singapore	Taipei	New York
**Washington**	New York	Los Angeles	San Francisco	London	Chicago
**Dusseldorf**	Sydney	Toronto	Amsterdam	Paris	Melbourne
**Moscow**	London	New York	Paris	Tokyo	Hong Kong
**Stockholm**	Amsterdam	Toronto	Copenhagen	Dusseldorf	Brussels
**Bangkok**	Singapore	London	New York	Sydney	Hong Kong
**Santiago**	Madrid	Milan	Tokyo	Zurich	Sao Paulo
**Buenos Aires**	London	Tokyo	New York	Zurich	Madrid
**Johannesburg**	London	New York	Hong Kong	Tokyo	Sydney
**Barcelona**	Madrid	Amsterdam	Paris	Brussels	Toronto
**Dallas**	Chicago	Washington	San Francisco	Toronto	New York
**Montreal**	Toronto	Dusseldorf	London	Sydney	Zurich
**Manila**	Sydney	Singapore	Taipei	London	Bangkok
**Warsaw**	Prague	London	Paris	Moscow	Singapore
**Kuala Lumpur**	Hong Kong	Singapore	Taipei	Sydney	London
**Prague**	Warsaw	London	New York	Istanbul	Singapore
**Caracas**	Tokyo	Sao Paulo	Zurich	Madrid	Milan
**Budapest**	Paris	Singapore	London	Tokyo	New York
**Beijing**	London	Sydney	Singapore	New York	Paris
**Geneva**	Frankfurt	Hong Kong	Tokyo	Singapore	New York
**Miami**	New York	Tokyo	London	Los Angeles	Mexico City
**Boston**	San Francisco	Dusseldorf	Los Angeles	London	Washington
**Istanbul**	Prague	London	Paris	New York	Dusseldorf
**Copenhagen**	Stockholm	Dusseldorf	Hamburg	Toronto	Amsterdam
**Hamburg**	Dusseldorf	Copenhagen	Munich	Toronto	Stockholm
**Rome**	Milan	Tokyo	Madrid	Toronto	Frankfurt
**Munich**	Hamburg	Dusseldorf	Toronto	Berlin	Sydney
**Houston**	Chicago	San Francisco	Washington	New York	Hong Kong
**Shanghai**	Seoul	Taipei	Kuala Lumpur	Bangkok	Singapore
**Berlin**	Munich	Washington	Hamburg	Dusseldorf	Toronto
**Atlanta**	Dallas	Chicago	Washington	Hamburg	Los Angeles
**Osaka**	Atlanta	Dusseldorf	Hamburg	Chicago	Munich
**Minneapolis**	Atlanta	Washington	Hamburg	Copenhagen	Dusseldorf

Appearances of top 5 weighted degree nodes as five nearest neighbours: London- 36 (11); New York- 34 (9); Paris- 20 (12); Tokyo- 20 (2); Hong Kong- 17 (6). In total, this makes 46.18% of the entire closest five relations.

Proximal relations: 139— 28.78% of which are due to top 5 weighted degree cities. 38 cities appear as nearest neighbours.

Table C. Five Nearest Neighbours (NN) for each node of the original World city network (Green indicates globally proximal neighbour)

City	NN1	NN2	NN3	NN4	NN5
**London**	New York	Paris	Hong Kong	Tokyo	Singapore
**New York**	London	Hong Kong	Paris	Los Angeles	Tokyo
**Paris**	London	New York	Tokyo	Hong Kong	Sydney
**Tokyo**	New York	London	Paris	Hong Kong	Singapore
**Hong Kong**	New York	London	Paris	Tokyo	Singapore
**Sydney**	London	New York	Paris	Hong Kong	Singapore
**Singapore**	London	New York	Hong Kong	Tokyo	Paris
**Milan**	New York	London	Paris	Tokyo	Madrid
**Frankfurt**	New York	London	Paris	Tokyo	Hong Kong
**Toronto**	London	New York	Paris	Sydney	Milan
**Madrid**	London	New York	Paris	Milan	Tokyo
**Brussels**	London	New York	Paris	Madrid	Milan
**San Francisco**	New York	London	Hong Kong	Los Angeles	Paris
**Los Angeles**	New York	London	Paris	Hong Kong	Washington
**Zurich**	London	New York	Tokyo	Paris	Frankfurt
**Mexico City**	London	New York	Tokyo	Paris	Hong Kong
**Chicago**	New York	London	Paris	Tokyo	Hong Kong
**Sao Paulo**	London	New York	Tokyo	Paris	Milan
**Amsterdam**	London	New York	Paris	Sydney	Toronto
**Taipei**	New York	London	Hong Kong	Paris	Sydney
**Melbourne**	London	New York	Sydney	Paris	Toronto
**Jakarta**	London	New York	Paris	Sydney	Tokyo
**Seoul**	London	New York	Paris	Sydney	Hong Kong
**Washington**	New York	London	Los Angeles	Paris	San Francisco
**Dusseldorf**	London	Paris	New York	Sydney	Toronto
**Moscow**	London	New York	Paris	Tokyo	Hong Kong
**Stockholm**	London	New York	Paris	Sydney	Toronto
**Bangkok**	London	New York	Hong Kong	Singapore	Paris
**Santiago**	London	New York	Tokyo	Paris	Madrid
**Buenos Aires**	London	New York	Tokyo	Paris	Hong Kong
**Johannesburg**	London	New York	Tokyo	Hong Kong	Sydney
**Barcelona**	London	Paris	New York	Madrid	Milan
**Dallas**	New York	London	Paris	Chicago	Toronto
**Montreal**	London	New York	Paris	Sydney	Toronto
**Manila**	London	New York	Sydney	Paris	Singapore
**Warsaw**	London	New York	Paris	Tokyo	Singapore
**Kuala Lumpur**	London	New York	Hong Kong	Sydney	Singapore
**Prague**	London	New York	Paris	Tokyo	Singapore
**Caracas**	New York	London	Tokyo	Paris	Milan
**Budapest**	London	New York	Paris	Tokyo	Hong Kong
**Beijing**	London	New York	Paris	Sydney	Singapore
**Geneva**	New York	London	Paris	Tokyo	Hong Kong
**Miami**	New York	London	Tokyo	Paris	Hong Kong
**Boston**	London	New York	Paris	Sydney	San Francisco
**Istanbul**	London	New York	Paris	Sydney	Hong Kong
**Copenhagen**	London	New York	Toronto	Sydney	Paris
**Hamburg**	London	New York	Toronto	Paris	Sydney
**Rome**	London	New York	Paris	Tokyo	Milan
**Munich**	London	New York	Paris	Sydney	Toronto
**Houston**	New York	London	Hong Kong	Tokyo	Paris
**Shanghai**	London	New York	Singapore	Hong Kong	Sydney
**Berlin**	London	New York	Paris	Tokyo	Sydney
**Atlanta**	New York	London	Paris	Chicago	Washington
**Osaka**	London	New York	Tokyo	Paris	Toronto
**Minneapolis**	New York	London	Washington	Toronto	Paris

Appearances of top 5 weighted degree nodes as five nearest neighbours: London- 54 (21); New York- 54 (14); Paris- 51 (20); Tokyo- 27 (4); Hong Kong- 24 (8). In total, this makes 76.64% of the entire closest five relations.

Proximal relations: 104—64.4% of which are due to top 5 weighted degree cities. Only 15 cities appear, including the top 11, with the rest made of Los Angeles, San Francisco, Chicago and Washington (all US).

References

[1] Watts D.J. & Strogatz, S. H. Collective dynamics of small-world networks. *Nature* **393,** 440–442 (1998).

[2] Estrada, E. Quantifying network heterogeneity. *Phys. Rev. E* **82,** 066102 (2010).

[3] Johnson, S., Torres, J. J., Marro, J. & Munoz, M. A. The entropic origin of disassortativity in complex networks. doi:10.1103/PhysRevLett.104.108702 (2010)

[4] Broido A., and Clauset A., "Scale-free networks are rare," Nature Communications, vol. 10, 1017 (2019).

[5] Voitalov I., van der Hoorn P., van der Hofstad R., and Krioukov D., "Scale-free networks well done," Physical Review Research, vol. 1, p. 033034, 2019.

[6] Strehl A. & Ghosh J., "Cluster ensembles- a knowledge reuse framework for combining multiple partitions", *Journal of Machine Learning Research*, 3: 583-617 (2003)
